# Effect of Pre-Vulcanization Time on Structure and Thermal Insulation of Natural Rubber Latex/Silica Aerogel Composites

**DOI:** 10.3390/gels12070599

**Published:** 2026-07-05

**Authors:** Chayanan Boonrawd, Wanwilai Vittayakorn, Darapond Triampo, Supan Yodyingyong

**Affiliations:** 1 Department of Nanoscience and Nanotechnology, School of Integrated Innovative Technology, King Mongkut’s Institute of Technology Ladkrabang, Bangkok 10520, Thailand; wanwilai.vi@kmitl.ac.th; 2 Department of Chemistry, Faculty of Science, Mahidol University, Nakhon Pathom 73170, Thailand; darapond.tri@mahidol.edu; 3 Institute for Innovative Learning, Mahidol University, Nakhon Pathom 73170, Thailand; supan.yod@mahidol.edu

**Keywords:** silica aerogel, natural rubber latex, pre-vulcanization, thermal insulation materials

## Abstract

Polymer/Silica aerogel (SA) composites improve mechanical properties strategically, but the mixing process disrupts the aerogel’s structure, reducing its efficiency due to polymer chains filling the pores. Pre-vulcanized natural rubber latex (PVNRL) with a higher crosslink density can strain the moving chains, thereby preserving the SA-porous structure in the bulk composite for thermal insulation materials. This study aimed to investigate the effects of PVNRL pre-vulcanization time and SA-immersion time in PVNRL. For PVNRL/SA composite preparation, various PVNRL, from 0 days to 8 days of pre-vulcanization time, were mixed with a fixed SA content of 20 parts per hundred of rubber (phr) using a latex compounding method. Subsequently, the PVNRL/SA slurries were cast on glass plates with 0, 3, and 6 days to obtain the PVNRL/SA composite. Considering the effect of pre-vulcanization time, the crosslink density of the composite increased and revealed a peak at PVNRL/SA with 8-day PVNRL by 7.277 ± 0.881 μmol $g^{-1}$, corresponding to the closest percentage of pore area in the SA’s structure to the pristine SA, and eventually a 42.41% lower thermal conductivity than the PVNRL/SA with 0-day PVNRL exhibited. In addition, the thermal conductivity increased more slowly over immersion time with the presence of 8-day PVNRL. The proposed correlation states that increasing the pre-vulcanization improves the thermal insulation performance of PVNRL/SA composites, emphasizing the reduction of filled SA’s pore with unvulcanized NR chains. Furthermore, the PVNRL/SA composite with 8-day PVNRL maintains thermal stability at 387.3 °C, and can be flexed at room temperature. These fascinating discoveries may be advantageous for further applications related to thin-film and flexible thermal insulation materials.

## 1. Introduction

Thermal insulation has been a promising solution for reducing energy consumption worldwide by preventing unnecessary heat energy loss and gain from buildings, factories, and thermal control systems. The cutting-edge development of thermal insulation materials has continued, especially through the use of aerogel constituents. Silica aerogel (SA) is a remarkable lightweight thermal insulation material. The exceptional porous structure of the SA comprises nanospherical particles in agglomerated, necklace-like formations. These interweaving necklaces form several open nanometric pores, ranging in size from a few to tens of nanometers [1,2]. Due to their unique structure, they exhibit a porosity range from 75% to 99% and a notably high specific surface area. These features bring about the lowest density of solid materials and the lowest thermal conductivity within the range from 0.01 to 0.03 W m^−1^K^−1^ [3,4,5,6]. In brief, the heat-insulating mechanism of SA is achieved by confining nanometric air within the aerogel pores and then forming chambers to minimize heat transfer between gas molecules. Consequently, this inhibits air convection and reduces the gas-phase thermal conductivity [1,7,8]. However, brittleness and fragility due to SA’s properties, such as extremely low density and high porosity, pose significant challenges for the use of SA monoliths [9,10].

To overcome the problems, a composite of a polymer and SA (polymer/SA composite) is suggested as a promising approach to improve SA properties [11,12,13]. The advantages of polymer/SA composites include not only enhanced mechanical properties but also synergistic improvements in polymer properties that align with market trends [14,15], for instance, polyimide to increase high heat resistance in the bulk composite [16], epoxy for improvement of the temperature range in utilization [17], and elastomer for an increase in elasticity [18,19,20]. However, the inevitable issue in preparing the polymer/SA composite is SA’s pore impregnation by the polymer chains, which reduces the composite’s thermal insulation properties.

Consequently, the investigation of SA’s pore preservation for polymer/SA has been continued and reported [21]. Lee H. et al. prepared the composite between SA and poly(dimethylsiloxane) through pre-mixed ethanol and SA prior to combining with pre-polymer, and eventually adding the hardener to fix the polymer chains before the drying process [18]. Boonrawd C. et al. premixed SA with cyclohexane prior to mixing with natural rubber latex (NRL), resulting in an oil-in-water slurry that formed a solvent barrier, preventing infusion between SA and NRL during the drying process [22]. Choi. W. S. et al. reported the SA pore preservation by adjusting the viscosity of polypropylene (PP) via polymer chain length; the higher viscosity of PP increased the performance of SA’s pore preservation [21]. According to previous research, pore-preservation approaches without chemical modification fall into two categories. First, they utilize the low-surface-tension solvents such as ethanol [17], hexane [20], and cyclohexane [22] to premix with SA prior to polymer introduction. Because these solvents evaporate more readily, they can prevent SA collapse during drying, which is triggered by repulsion between the alkyl group from the aerogel synthesis, called the spring-back effect [23]. Second, limiting polymer movement during mixing is achieved by crosslinking [18] or by using higher-molecular-weight (higher viscosity) polymers [21,24] before they contact the SA pores. To adopt a more eco-friendly approach by reducing the use of organic solvents, only the second way would be continued. Therefore, a suitable polymer matrix becomes an interesting issue.

The colloidal biopolymer (poly(1,4-cis-isoprene)) produced naturally by the rubber tree (*Hevea brasiliensis*) consists of rubber particles (RPs) dispersed in water, in the form of NRL [25,26,27,28]. The RPs consist of coiled-up rubber chains within a spherical structure, with localization of natural surfactants, i.e., phospholipids and proteins. This structure plays a significant role in hindering the interparticle diffusion of rubber chains after water evaporation and close packing of RPs during film formation [29]. The distinguished properties of the NR exhibit excellent mechanical and dynamic properties, elasticity, and flexibility [30]. However, the presence of C=C bonds in the chemical structure of NR serves as the active site for the oxidative reaction, leading to chain scission and, eventually, diminished mechanical properties. To address this dilemma, the double bonds are sacrificed during vulcanization, which is achieved via various methods such as sulfur, peroxide, and radiation systems. Pre-vulcanization is generated for crosslinking in the latex form, and the crosslinking agents are compounded in the liquid-like stage. The mechanism of pre-vulcanization using sulfur as a crosslink agent kicks off at the RP’s surface into the core of RPs, corresponding to sulfur diffusion. In addition, the unvulcanized NR chains are impeded between RPs during film formation with increased pre-vulcanization time from 0 to 8 days [31]. Hence, the PVNRL has the potential to diminish the unvulcanized chains’ ability to move and fill the SA’s pores during the mixing and drying stage, due to its natural RP structure and pre-vulcanization.

This work aims to study the effect of pre-vulcanization on the thermal insulation performance of PVNRL/SA composites, following the hypothesis that reducing free-moving chains by pre-vulcanization helps retain SA’s porous structure, thereby affecting thermal insulation performance. PVNRL/SA composites with different pre-vulcanization times, as well as composites with different immersion times of SA in the mixture, are prepared. The SA content is fixed at 20 parts per hundred rubbers (phr), the maximum amount for latex compounding, as reported previously [32]. The thermal insulation performance, as indicated by thermal conductivity, is studied as a function of pre-vulcanization time. The physico-properties of the composites are investigated by crosslink density and microstructure from Field-emission scanning electron microscopy (FE-SEM) images to depict the reduction performance of the free-moving chains and preserve the nano-pore of SA, respectively. In addition, the correlation between thermal conductivity and crosslink density is proposed, along with studies of the thermal stability and flexibility of potential PVNRL/SA composites as satisfactory future thermal insulation materials.

## 2. Results and Discussion

### 2.1. Chemical Composition Analysis

**Figure 1:**
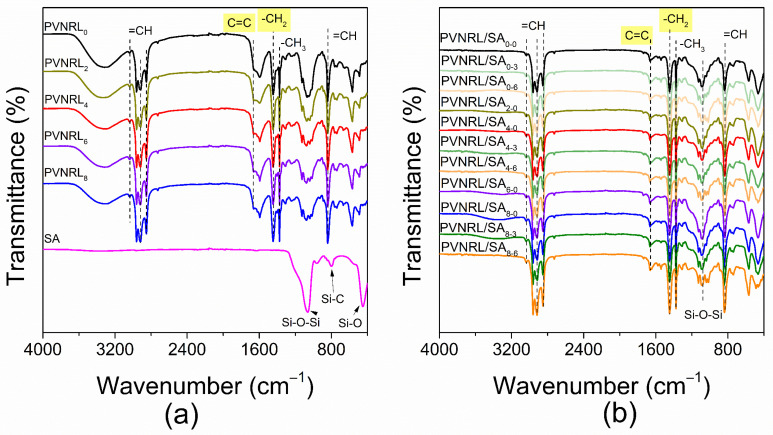
FT-IR spectra of (**a**) PVNRL-controlled samples with different pre-vulcanization times and SA, (**b**) PVNRL/SA composite using 0, 2, 4, 6, and 8-day PVNRL, and the PVNRL/SA composites using 0-, 4-, and 8-day PVNRL in different immersion times by 3 and 6 days.

The chemical composition of the PVNRL/SA composites and their immersion series with different pre-vulcanization times was confirmed by a Fourier transform infrared spectroscopy (FT-IR), with PVNRL samples and SA as references, as shown in Figure 1. To properly understand, the sample names were labeled PVNRL/SA_x-y_, where x and y refer to the pre-vulcanization and immersion times, respectively.

The absorption bands, as presented in Figure 1a, revealed the principal characteristic bands of NR, indicative of poly(cis-1,4-isoprene), at 3031, 2915, and 2850 ${\mathrm{cm}}^{-1}$, corresponding to symmetric and asymmetric stretching of =CH bonds. The peak at 1662 ${\mathrm{cm}}^{-1}$ indicated the stretching vibration of C=C bonds, whereas the peaks at 1446 and 1371 ${\mathrm{cm}}^{-1}$ are referred to the deformation of –CH_2_ and –CH_3_ groups, respectively. Moreover, the band at 839 ${\mathrm{cm}}^{-1}$ corresponds to the bending of =CH bonds located outside the molecular plane, ascribing the presence of cis-1,4 [33,34]. In addition, the adsorption peaks, referred to as SA, at 1073, 789, and 451 ${\mathrm{cm}}^{-1}$, ascribed different modes of Si–O–Si bending, Si–C, and Si–O stretching vibration, respectively [28]. As presented in Figure 1b, all corresponding bands referring to NR were observed in the FT-IR spectra of all PVNRL/SA composites. Additionally, the characteristic band of SA, namely Si–O–Si, was exhibited as broad peaks in the PVNRL/SA spectra. Therefore, it can be stated that the existence of the NR and SA in the composites is confirmed.

**Figure 2:**
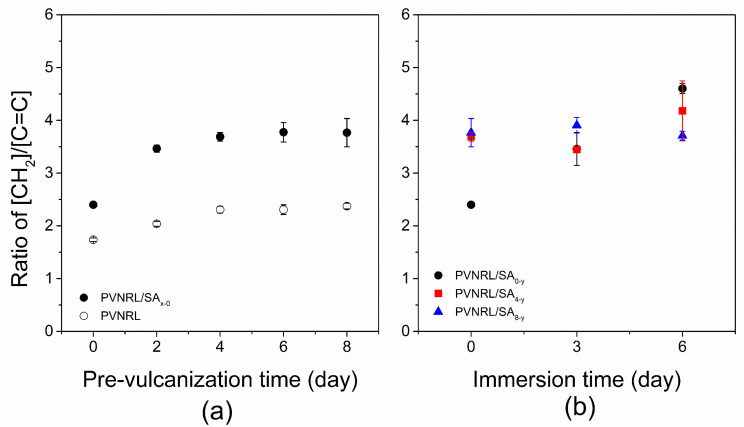
FT-IR intensity ratios between –CH_2_ and C=C of (**a**) PVNRL-controlled samples, PVNRL/SA composites with different pre-vulcanization times, and (**b**) PVNRL/SA composites 0-, 4-, and 8-day PVNRL in different immersion times (as noted that x and y refer to pre-vulcanization times, and immersion times, respectively).

Since the C=C in NR chains would interact with the sulfur curing agent by the pre-vulcanization process, the crosslinking activities of the NR chains were considered roughly via the decrease in the band intensity of C=C bonds at 1662 ${\mathrm{cm}}^{-1}$ [35]. This indirect evidence was obtained through the comparison of the band intensity between the unchanged functional group in NR chains, i.e., –CH_2_ at 1446 ${\mathrm{cm}}^{-1}$, and the double bond band. The increment of the ratios means the disappearance of the proportion of C=C in the composites. The intensity ratios of all PVNRL/SA composites and their controlled samples (PVNRL samples) were examined, as presented in Figure 2.

Considering the effect of pre-vulcanization time as shown in Figure 2a, the intensity ratios between –CH_2_ and C=C of PVNRL/SA increased significantly with increasing pre-vulcanization time from 0 to 2 days, from 2.399 ± 0.039 to 3.463 ± 0.069 (approximately 30.72%). Then, a gradual rise was detected, reaching its plateau in PVNRL/SA_6-0_ (3.774 ± 0.185) and PVNRL/SA_8-0_ (3.765 ± 0.268), an increase of over 59.93% from the beginning of pre-vulcanization. Similar behavior appeared in the PVNRL-controlled samples, and exhibited lower ratio values than those of the composites with the existence of SA. These results suggest that crosslinking in NR via the sacrificed C=C bonds increases with pre-vulcanization time and the presence of SA.

To consider the immersion time effect, the PVNRL/SA composites using 0, 4, and 8 days of PVNRL were investigated through the different immersion times of 0, 3, and 6 days, and the intensity ratio of these samples is shown in Figure 2b. Using 0-day PVNRL, the intensity ratio increased by about 0.91 times with immersion time. Subsequently, the intensity ratios versus immersion time for the composites using 4-day and 8-day PVNRL showed slight changes, with maximum values in a similar range (over 3.400), reflecting greater scission of the C=C bonds in the NR chains. In addition, the slow increase in the intensity ratio at the no-immersion stage (Figure 2a) may be attributed to reaching the limited point to crosslink of NR chains at the surface of RPs. Hence, it may be assumed that PVNRL/SA composites with longer pre-vulcanization times may achieve sufficiently high crosslink densities to confine free-moving chains in the PVNRL/SA mixture, independent of immersion time.

### 2.2. Crosslink Density

**Figure 3:**
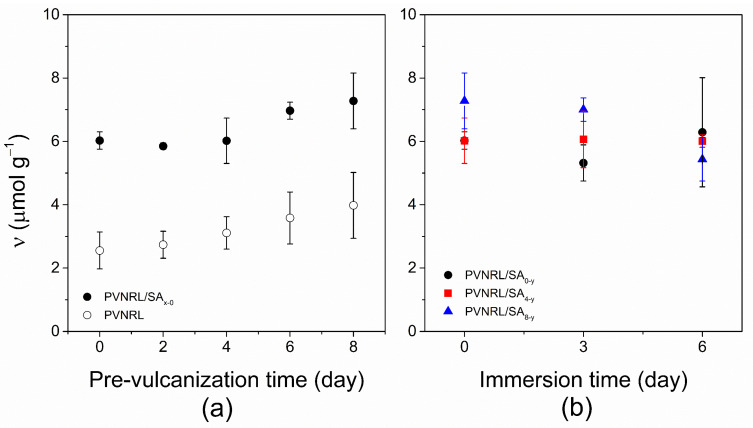
Crosslink density of (**a**) PVNRL-controlled samples, PVNRL/SA composites with different pre-vulcanization times, and (**b**) PVNRL/SA composites 0-, 4-, and 8-day PVNRL in different immersion times (as noted that x and y refer to pre-vulcanization times, and immersion times, respectively).

Crosslink densities (ν) of the PVNRL/SA composites with different pre-vulcanization times, including their PVNRL-controlled samples, are presented in Figure 3a. It can be seen that the PVNRL/SA composites showed a higher degree of crosslinking than their pristine counterparts without SA, with the synchronized pre-vulcanization times. Considering the pre-vulcanization time effect, the ν-values of the pre-NR increased with pre-vulcanization time, corresponding to the FT-IR results from 2.555 ± 0.580 μmol $g^{-1}$ to 3.979 ± 1.040 μmol $g^{-1}$. Alternatively, a slight change in the ν-values was observed in the PVNRL/SA composites using lower 4 day-PVNRL, followed by a rapid increase beyond 4 days, and continued to increase slightly until 8 days of pre-vulcanization from 6.025 ± 0.272 μmol $g^{-1}$ to 7.277 ± 0.881 μmol $g^{-1}$. It was approximately 1.84 times from the 0-day in pre-vulcanization time.

These behaviors may be described by the pre-vulcanization mechanism in latex form and by the NR chain at RPs during film formation. First, the diffusion of the curing agents from the surface to the inside chains in RPs depends on time. Therefore, PVNRL with a longer pre-vulcanization time results in a greater number of curing agents penetrating the core of RPs, leading to a higher NR-NR crosslink density. As evidenced in PVNRL-controlled samples, the crosslink density continued to rise with increasing pre-vulcanization times. Second, the status of NR chains during film formation reflects the entanglement ability of unvulcanized NR chains, resulting in NR-SA physical crosslinks at the PR boundaries. The PVNRL/SA composites using PVNRL below 4 days demonstrated a lower crosslink density, attributed to higher unvulcanized NR chains. The NR-SA interaction of these composites would dominate during the compaction of the RPs from water evaporation during film formation, resulting in a slight change and a higher crosslink density than in the PVNRL-controlled samples. Furthermore, NR-NR crosslinks reverse to influence, and slightly decline in the entangle of NR-SA. This explained that the PVNRL/SA composite with PVNRL longer than 4 days displayed higher ν-values with increasing pre-vulcanization time, but there was a smaller difference in ν-values between the composites and their PVNRL-controlled samples than between the composites with shorter pre-vulcanization times. Consequently, the increase in pre-vulcanization times assists in reducing the unvulcanized chains to form NR-SA entanglements during film formation.

As shown in Figure 3b, the crosslink densities of PVNRL/SA composites with immersion times of 0, 3, and 6 days exhibited distinct behaviors. The PVNRL/SA using 0 and 4 days of PVNRL showed ν-values in the NR-SA crosslink-dominant range (5.319 ± 0.571–6.287 ± 1.723 μmol $g^{-1}$), suggesting that this behavior reflects a slight change in NR-NR crosslinks over immersion time. In other words, the decline in ν-values of PVNRL/SA using 8-day PVNRL was observed: a gradual decrease at 3 days (7.004 ± 0.370 μmol $g^{-1}$) and a rapid fall at 6 days (5.432 ± 0.687 μmol $g^{-1}$) of immersion time. The complicated mechanism can be ascribed to the hard-shell/soft-core effect for PVNRL over 8 days, which relates to the difficulty of curing agents diffusing into the core of PRs due to the high crosslink density at the surface of RPs, leading to lower crosslink density, as it should be, in the chains inside PRs, and to the broken boundary of RPs during film formation [31]. It seemed that NR-NR crosslinks influenced and cooperated with the slow formation of NR-SA interactions because of the notably low unvulcanized chains at the beginning; therefore, the overall crosslink density of the bulk composites decreased with immersion time, reflecting the inclination of NR-NR crosslinks. To summarize, the pre-vulcanization times affect the crosslink density of PVNRL/SA and indicate the optimal time to achieve and maintain a high crosslink density throughout immersion.

### 2.3. Morphology

**Figure 4:**
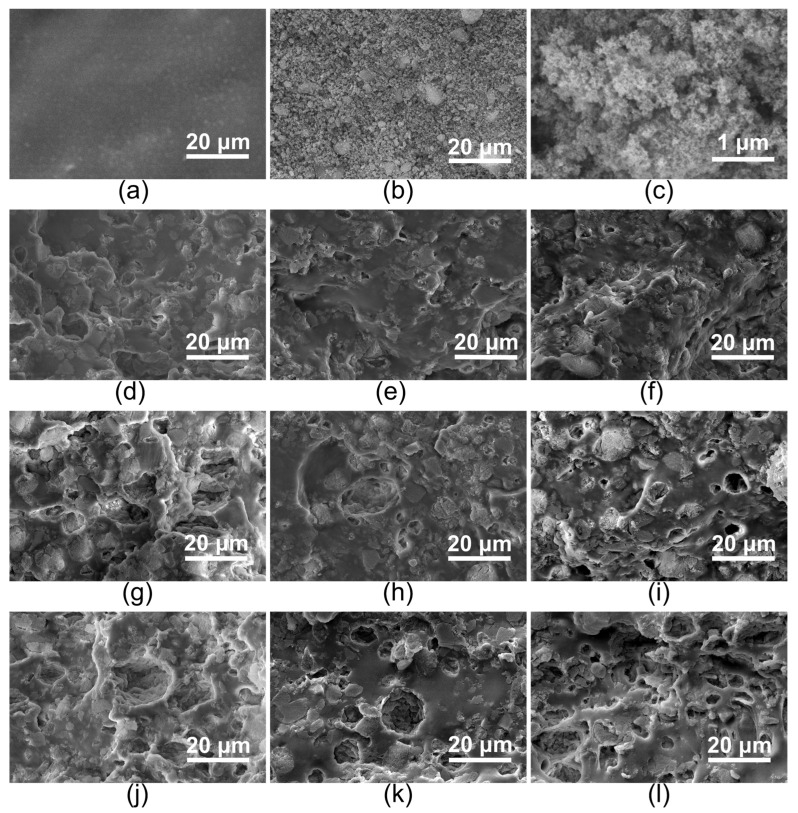
Cross-section FESEM images of (**a**) PVNRL_0_-controlled sample (at ×5K magnification), SA with different magnifications as (**b**) ×5K and (**c**) ×100K, and the low magnification FE-SEM images (×5K) of PVNRL/SA composites using 0-day PVNRL in different immersion times as (**d**) 0 day, (**e**) 3 days, and (**f**) 6 days, including PVNRL/SA using 4-day PVNRL in various immersion times as (**g**) 0 day, (**h**) 3 days, and (**i**) 6 days, and likewise, PVNRL/SA using 8-day PVNRL in similar range of immersion times as (**j**) 0 day, (**k**) 3 days, and (**l**) 6 days.

The cross-sectional morphologies of PVNRL/SA composites were imaged by FE-SEM, indicating the consequences of the pre-vulcanization performance to limit the freely movable chains for preserving the SA’s porous structure. The dark, smooth region represented the NR (Figure 4a), and the fluffy particle structure represented the SA (Figure 4b) at low magnification, serving as references for assessing the bulk microstructure of PVNRL/SA composites with different pre-vulcanization times, including those with various immersion times. In addition, the porous structure of SA was exhibited in the high magnification images (Figure 4c) for the study of SA’s pore preservation. As presented in Figure 4d–l, the PVNRL/SA composites prepared with different pre-vulcanization times presented the existence of both NR and SA, which possessed a heterogeneous pore structure, consisting of macroscale pores and SA’s nanoscale pores.

The macroscale pore structure of PVNRL/SA composites showed a range of pore sizes. In addition, the PVNRL/SA_0-0_ composites appeared to be the lowest among the pores when compared to the PVNRL/SAPVNRL/SA_4-0_ and PVNRL/SA_8-0_ composites, as shown in Figure 4g and Figure 4j, respectively. To clearly understand the effect of pre-vulcanization time, the area and diameter of the pores in the bulk PVNRL/SA composites were determined in a similar investigated area of 5K magnification-FFSEM (82.69 × 55.13 ${\mu m}^{2}$), as presented in Table 1. The increase in pore area was observed, approximately 1.51 times, with an increase in pre-vulcanization time, and reached the peak at PVNRL/SA_8-0_ by 23.391 ± 2.001%. The microstructures of the PVNRL/SA with different immersion times were repeated. With increasing immersion times, the PVNRL/SA using 0-day PVNRL (Figure 4e,f) showed a slight difference in pore area percentage of approximately 1%, and a more compact structure was observed than in other PVNRL/SA composites. Notable declines in pore area percentage over immersion time were observed in PVNRL/SA composites with 4 days (Figure 4h,i) and 8 days (Figure 4k,l) of pre-vulcanization time, resulting in decreases of 4.385% and 6.620% from the no-immersion day composites, respectively. In the discussion, the consequence results are related to the free-moving chain limitation imposed by the crosslink density of the PVNRL/SA composites, which increased with increasing pre-vulcanization time. The observed empty macropores and SA-filled macropores were generated by lower entanglement of NR-SA during film formation, leading to individual locations between the SA and NR phases until the composites dried. Interestingly, as shown in Table 1, the average pore diameter of the PVNRL/SA composites increased gradually (more than 8%) with broader error adjustment over the pre-vulcanization time. This behavior is related to the lower NR-SA entanglement from lower unvulcanized NR chains, leading to polydispersity in pore creation due to insufficient movable chains to penetrate imperfectly into the SA-filled pores. Considering the immersion time effect, the PVNRL/SA using 0-day PVNRL showed a slight decrease in pore diameter (less than 9%) (with increasing immersion time, with the narrower error adjustments of less than 3 μm. A noticeable change in pore diameter was observed in PVNRL/SA using 4-day PVNRL, with a decline of 22.87% as the immersion time increased, akin to the error adjustments (≥0.5 μm-decline). It can be attributed to the longer immersion time, which results in greater contact between unvulcanized NR chains and SA at the pore boundary and leads to higher NR-SA entanglement, sufficient to narrow the pore diameter of the PVNRL/SA composites. The unusual macropore generation was observed by the pore diameter of PVNRL/SA composite using 8-day PVNRL. The various pore diameters presented in the range from 6.175 ± 3.832 to 7.108 ± 6.792 μm with broad error adjustments. This result confirmed the efficiency of the high NR-NR crosslinks in 8-day PVNRL, which led to the formation of larger pores within the composites as the immersion time increased. However, the unvulcanized NR chains resulting from the hard-shell/soft-core effect still appeared through the shift down in PVNRL/SA_8-6_’s pore diameter and narrower err adjustments. Accordingly, it can be summarized that increased pre-vulcanization of PVNRL can generate a higher pore area percentage and larger pore diameter, indicating a proper macropore structure for thermal insulation in the PVNRL/SA composite.

**Figure 5:**
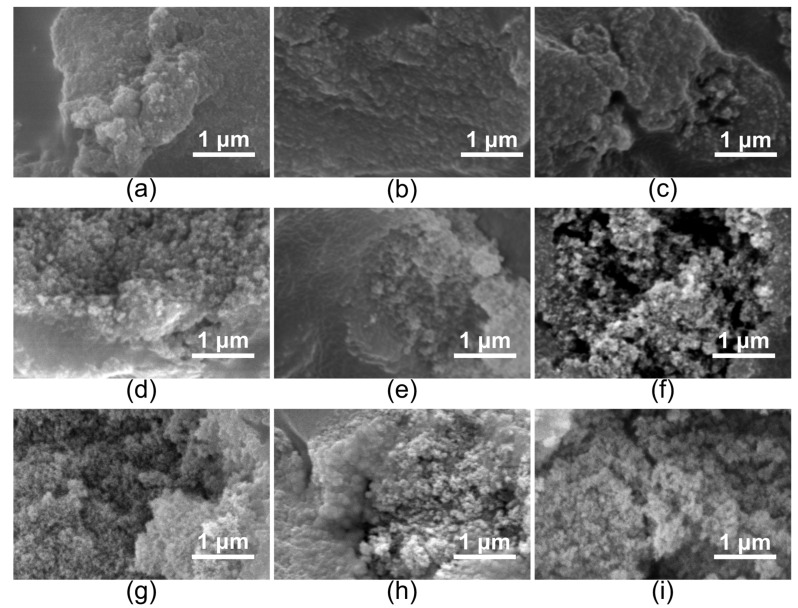
Cross-section high magnification FESEM images (×100K) of PVNRL/SA composites using 0-day PVNRL in different immersion times, as (**a**) 0 day, (**b**) 3 days, and (**c**) 6 days, including PVNRL/SA using 4-day PVNRL in various immersion times as (**d**) 0 day, (**e**) 3 days, and (**f**) 6 days, and likewise, PVNRL/SA using 8-day PVNRL in similar range of immersion times as (**g**) 0 day, (**h**) 3 days, and (**i**) 6 days.

The high magnification of PVNRL/SA composites to express the existence of SA’s porous structure, thanks to the pre-vulcanization times, is presented in Figure 5. Compared to the pristine porous structure of SA (Figure 4c), the compact structure presented for SA in PVNRL/SA with 0 days of pre-vulcanization, as that of its immersion composites, is shown in Figure 5a–c. The tiny pores observed in the PVNRL/SA composites using 4- and 8-day PVNRL served as a signature of the aerogel structure, as shown in Figure 5d–i. A qualitative study to supportively describe the microstructure of the PVNRL/SA composites was conducted by evaluating the area and diameter of SA’s pores. The 100K magnification-FFSEM (4.12 × 2.75 ${\mu m}^{2}$) was used as the investigated area, and the data are exhibited in Table 2. The almost undetectable pore area and diameter were observed in PVNRL/SA using the 0-day PVNRL series, similar to those in composites with different immersion times. This behavior is attributed to a higher number of unvulcanized free-movable chains, resulting from the lower crosslink density, leading to higher NR-SA entanglements and SA’s pore impregnation. The observed porous structure of SA in PVNRL/SA_4-0_ composites corresponds to the significant increase in pore area by 19.198 ± 1.999% then, gradually decrease (≤2.5%) with immersion time. In the same way, the PVNRL/SA8-0 revealed the highest pore area of 29.480 ± 7.623%, which is closest to that of pristine SA, and subsequently declined to below 20% with increasing immersion time.

These behaviors can be described correspondingly by the assistance of higher crosslink PVNRL to the remaining of the SA’s porous structure, by reducing unvulcanized NR chains. The pore diameter attributed to the interspace between SA’s skeleton was slightly decreased with an increase in pre-vulcanization time from 4 days to 8 days, reaching the lowest pore diameter by 0.090 ± 0.034 μm. In other words, the increment of pore diameter was observed over immersion time in each PVNRL series. These results suggest that the higher NR-SA entanglement, induced by either shorter pre-vulcanization or longer immersion time, led to denser SA agglomeration, resulting in a broader interspace between the SA skeleton and, consequently, a larger pore diameter observed. Thus, it can be implied that using higher crosslink PVNRL with increasing pre-vulcanization times before mixing with SA improves the retention of SA’s porous structure by reducing the unvulcanized free-movable NR chains.

**Figure 6:**
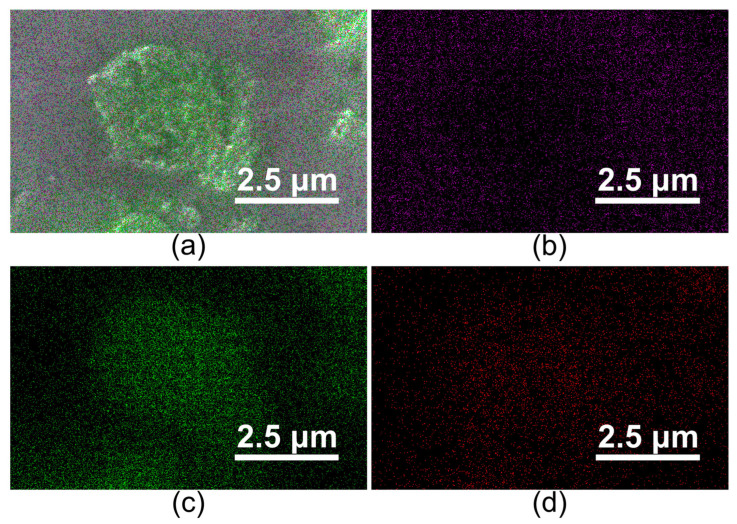
(**a**) EDX images of the PVNRL/SA_8-0_ composite with component signals consisting of (**b**) carbon, (**c**) silicon, and (**d**) oxygen.

The composition of the PVNRL/SA_8-0_ composites was reconfirmed by the energy dispersive X-ray spectroscopy (EDX) images, as shown in Figure 6a. The observed carbon (C) signals in purple dots (Figure 6b) represent the NR, localized as a polymer matrix around the pores, with the apparent silicon (Si) and oxygen (O) signals in red (Figure 6c) and green dots (Figure 6d), respectively, referring to the SA elemental composition. The low intensity of the C signals was mainly observed in the macropore containing the SA, suggesting that partially moving NR chains were present during preparation and that the methyl group (–CH_3_) was from the hydrophobic surface modification of the aerogel [36]. These phenomena contributed to the effective limitation of the movable chains of NR during the pre-vulcanization period, helping preserve the aerogel structure and improve the thermal insulation performance of the composite.

### 2.4. Thermal Insulation Performance

**Figure 7:**
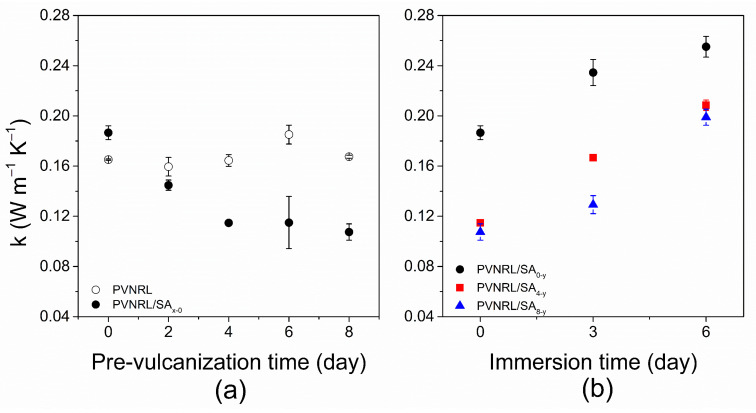
Thermal conductivities (k) of (**a**) PVNRL-controlled samples, PVNRL/SA composites with different pre-vulcanization times, and (**b**) PVNRL/SA composites 0-, 4-, and 8-day PVNRL in different immersion times (as noted that x and y refer to pre-vulcanization times, and immersion times, respectively).

Thermal insulation performance of the PVNRL/SA composites and the control samples prepared with different pre-vulcanization times of PVNRL is indicated by the decrease in thermal conductivity (k), as shown in Figure 7a. The k-values of the PVNRL/SA composites significantly decreased with increasing pre-vulcanization times from 0 days to 4 days, from 0.1866 ± 0.0055 to 0.1147 ± 0.0004 W m^−1^ K^−1^. Then, the values reached a plateau after 4 to 6 days, and showed the lowest k-value presented at the PVNRL/SA composites using 8-day PVNRL by 0.1074 ± 0.0064 W m^−1^ K^−1^, which could be approximately 42.41% lower than that of the PVNRL/SA with 0 days of pre-vulcanization time, corresponding to the microstructure and the observed highest pore area. Furthermore, the k-values of the PVNRL/SA_8-0_ declined by about 35.80%, whereas the PVNRL/SA_0-0_ showed a slight increase in k-values compared to their controlled PVNRL_8_ and PVNRL_0_. The reduction in thermal conductivity in this study was superior than that reported in previous work, which reported a 15% decline from the pristine NR sheet using the corresponding latex mixing method and SA contents of 20 phr [32]. Consequently, it could be stated that the pre-vulcanization times affect the thermal insulation performance of the PVNRL/SA composites.

The effect of immersion time on thermal insulation performance was investigated using the PVNRL/SA with immersion times of 0, 3, and 6 days, as shown in Figure 7b. The k-values of all representative PVNRL/SA composites increase with increasing immersion time, suggesting stronger NR-SA interactions during storage of the PVNRL/SA. The significantly lower k-values across all immersion times were observed in the PVNRL/SA composite using 4- and 8-day PVNRL, compared to those prepared with 0-day PVNRL. This evidence is parallel with the presence of a porous structure of the composites. Furthermore, the PVNRL with a pre-vulcanization time of 8 days showed the lowest k-values for its PVNRL/SA composite at all immersion times. At an immersion time of 3 days, the slower increase in k-values observed in PVNRL/SA_8-3_ composites from the initiation day, when compared to the composite prepared with other PVNRL, was interesting. Consequently, it can be implied that the thermal insulation of PVNRL/composites with different immersion times and the retardance of the thermal conductivity increment are related to the pre-vulcanization times of PVNRL.

**Figure 8:**
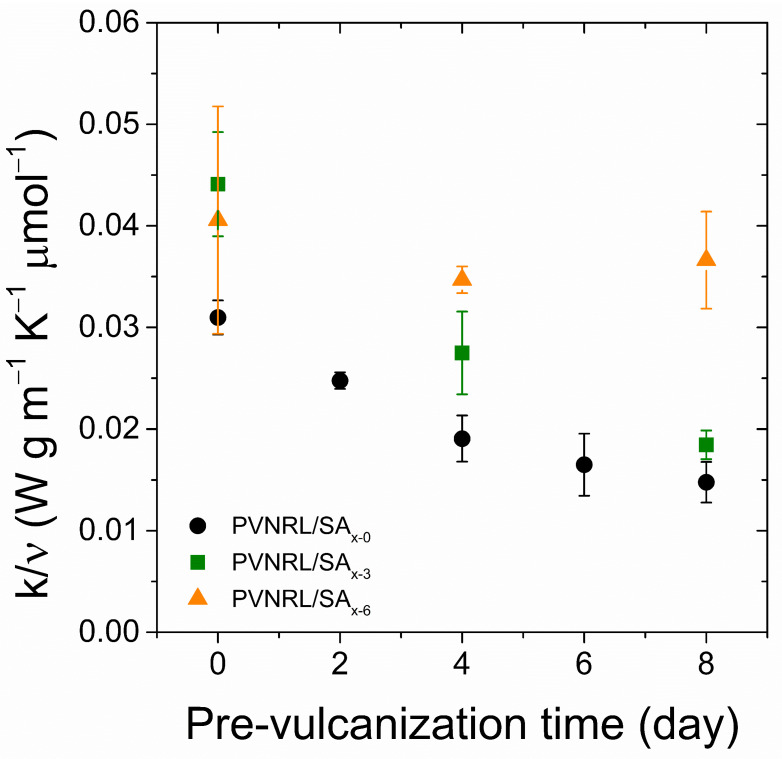
The correlation between pre-vulcanization time, thermal conductivity (k), and crosslink density (ν) of all PVNRL/SA composites with different pre-vulcanization times and immersion times (as noted that x refers to pre-vulcanization times).

To discuss and answer the hypothesis of this study, the correlation between pre-vulcanization time, thermal conductivity, and crosslink density was precisely studied to understand the mechanism of thermal insulation behavior in the PVNRL/SA composites. As illustrated in Figure 8, the divided results for the k- and ν-values of all PVNRL/SA composites, plotted against pre-vulcanization time, showed a downward trend for most composites. It can be observed that lower k-values are inversely related to ν-values and the pre-vulcanization times. This correlation supports the expected mechanism: the increase in pre-vulcanization time for PVNRL before SA mixing led to high NR-NR crosslinks in RPs, reducing the number of unvulcanized free-movable chains that could penetrate into SA’s porous structure during coalescence and film formation, and generating a porous structure on a micro-scale. Thereby, a thermal insulation path within the aerogel is reserved, including one in the bulk composites, resulting in lower thermal conductivity. The reverse trend is observed in PVNRL/SA_8-6_, suggesting that excessively long pre-vulcanization times increase unvulcanized NR chains during film formation due to the hard-shell/soft-core effect, as evidenced by lower crosslink density. Higher NR-SA interfacial entanglements compared to the original PVNRL reflect the higher SA’s filled pores, leading to destruction of the aerogel insulation pathway and, eventually, an increase in thermal conductivity. According to the correlation, the thermal insulation performance of PVNRL/SA composites inversely correlates with PVNRL’s pre-vulcanization time, and 8 days of pre-vulcanization is optimal for achieving the best thermal insulation performance.

### 2.5. Thermal Stability

**Figure 9:**
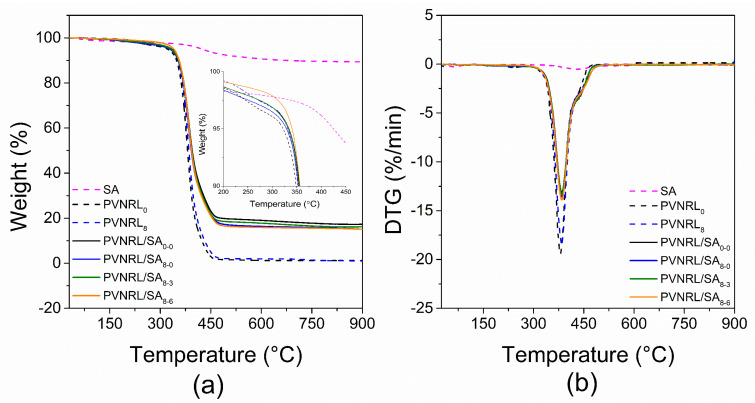
(**a**) TGA and (**b**) DTG thermographs of SA, PVNRL-controlled samples with 0- and 8-day pre-vulcanization time as the referenced samples, including PVNRL/SA composites 0- and 8-day PVNRL in different immersion times by 3 and 6 days.

Now, to investigate the thermal properties of the higher-potential composites to be satisfactory thermal insulation materials, thermal stability is crucial for future applications. As presented in Figure 9a,b, thermogravimetry analysis (TGA) and differential thermal analysis (DTG) thermograms of PVNRL/SA composites using 0-day and 8-day PVNRL showed a single typical weight loss at 300–450 °C and DTG peaks (approximately 383.6–384.0 °C) attributed to the C–C of NR segment decomposition [37], as observed in the PVNRL samples with each similar pre-vulcanization time. In addition, the slight weight loss of SA occurred at 385.5–461.8 °C, indicating the oxidative decomposition of the surface organic modifier (Si–CH_3_), leaving about 89.42% of the original residue. As evidenced by the heat-resistant residues at 900 °C in the PVNRL/SA composites, with a value of 16.55 for the residual existence, akin to pristine SA, it can be confirmed that a similar amount of SA was present in the composites. Considering the effect of pre-vulcanization time, the PVNRL/SA_8-0_ revealed a slightly lower decomposition temperature (T_d_) than PVNRL/SA_0-0_ and PVNRL_8_. Moreover, the PVNRL/SA_0-0_ showed T_d_ higher than PVNRL_0_, almost comparable to PVNRL_8_. These results may be described undeniably as the presence of SA and higher NR-SA interactions in PVNRL/SA_0-0_, meaning a rise in surface contact between the counterparts, and consequently an increase in the absorption of heat energy during decomposition [19]. In other words, the higher NR-SA interactions in the composite using 0-day PVNRL are due to the remaining unvulcanized movable NR chains. This chain mobility may destroy SA’s porous structure by filling it with NR chains, thereby reducing thermal conductivity. The micrograph of SA’s porous structure strongly supported this behavior. Therefore, the reasonable thermal stability of PVNRL/SA_8-0_ composite effectively supported its thermal insulation performance, and it is appropriate to investigate the effect of immersion time. The interesting thermal behavior of PVNRL/SA prepared by 8-day PVNRL with immersion times of 0 (no immersion), 3, and 6 days was demonstrated by a slight increase in T_d_ (from 387.3 °C to 387.9 °C) with increasing immersion time. It may be attributed to some small NR-SA crosslinks during immersion, because fewer unvulcanized NR chains were present, compared to the composite prepared with 0-day PVNRL.

### 2.6. Flexibility

**Figure 10:**
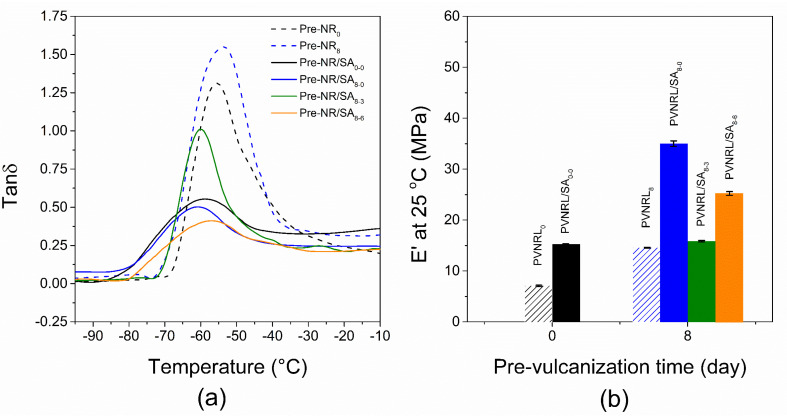
(**a**) Tanδ and (**b**) E’ at 25 °C of SA, PVNRL-controlled samples with 0- and 8-day pre-vulcanization time as the referenced samples, including PVNRL/SA composites 0- and 8-day PVNRL in different immersion times by 3 and 6 days.

Flexibility in this study is indicated by the glass transition temperature (T_g_), which was determined by a dynamic mechanical analysis (DMA) from the peak in loss tangent (Tan δ)- temperature correlation, as shown in Figure 10a. The T_g_ of all representative PVNRL/SA composites is composed of PVNRL/SA_0-0_, PVNRL/SA_8-0_ composites, and their PVNRL control samples for the study of pre-vulcanization time influence. In addition, the PVNRL/SA using 8-day PVNRL with immersion times of 0, 3, and 6 days was performed to investigate the effects of immersion time. The results exhibited that T_g_ for all PVNRL/SA composites shifted down to lower temperatures compared with the PVNRL control samples, approximately 2 and 6 °C differences for 0-day PVNRL and 8-day PVNRL without the immersion stage, respectively. It may be attributed to the lower NR-SA interaction presence when the pre-vulcanization time was increased. This would be additional evidence for preserving SA’s porous structure, in line with its microstructure and crosslink density, to reduce thermal conductivity. The interesting behavior observed in the PVNRL/SA composite using an 8-day PVNRL series with different immersion times showed a gradual increase in T_g_ (−60.85 to −56.85 °C) with increasing immersion time. It can explain that the stronger NR-SA interactions arise from the hard-shell/soft-core structure, as noted in the crosslink density results. Interestingly, the T_g_ of all represented PVNRL/SA composites was below room temperature, indicating that PVNRL/SA composites can exhibit high flexibility, similar to rubbery materials [18].

At a similar temperature with thermal conductivity evaluation (25 °C), the storage modulus (E’) of the PVNRL/SA_8-0_ was higher than that of PVNRL/SA_0-0_ by approximately 2 times, and the E’ of both composite corresponding higher value than their PVNRL with the same pre-vulcanization time, as displayed in Figure 10b. The following phenomena may suggest that the composite’s stiffness is mainly due to the NR crosslink and partially supported by NR-SA reinforcement, depending on the presence of free-moving NR chains. This reason can thereby explain the lower T_g_ of the composites than that of their control PVNRL. Therefore, it can be stated that the pre-vulcanization time played a crucial role in affecting the dynamic storage modulus of PVNRL/SA composites by increasing the degree of crosslinking. For the E’ of the 8-day PVNRL/SA series with different immersion times, a significantly lower E’ was observed in PVNRL/SA_8-3_ and gradually increased in PVNRL/SA_8-6_. This behavior agreed with their observed gradual increase in T_g_ of the composites with 3- and 6-day immersion times. It may be described that more unvulcanized NR chains were present because of the hard-shell/soft-core effect, and to synergism from higher NR-SA contacts over immersion time, leading to an increase in NR-SA interaction during film formation and an increase in T_g_. However, the 8-day PVNRL provided the NR crosslink sufficient time to retard the unvulcanized movable chains before SA was introduced, which not only improved SA’s pore preservation but also significantly affected E’ compared to NR-SA reinforcement, resulting in lower E’ observed in PVNRL/SA with longer immersion time.

## 3. Conclusions

The sustainable PVNRL/SA composites, prepared by PVNRL with different pre-vulcanization times and 20 phr of SA, were successfully fabricated by simple latex compounding. The challenged hypothesis is carried out by investigating the thermal insulation performance of the PVNRL/SA composite by adjusting only the pre-vulcanization time of the main component, PVNRL, from 0 to 8 days. In this study, the pre-vulcanization time directly affected the crosslink density and the FT-IR intensity ratios. The PVNRL/SA composites prepared with a higher crosslink density (i.e., 8 days) showed a porous structure in the bulk composite and the presence of SA’s pores, which is close to that of the pristine SA. It is related to the effective limitation of free-moving NR chains at higher crosslink density, thereby preserving SA’s porous structure. According to the potential physico-properties, the lowest thermal conductivity was presented in the PVNRL/SA_8-0_ by 0.1074 ± 0.0064 W m^−1^ K^−1^, 42.41% lower than the PVNRL/SA_0-0_, and 35.80% lower than its PVNRL_8_. Furthermore, PVNRL/SA composites prepared with 8-day PVNRL and varying immersion times showed a slower increase in thermal conductivity. All of these reasonable results generated the mechanism state that the thermal insulation performance of PVNRL/SA composites inversely correlates with PVNRL’s pre-vulcanization time, and 8 days of pre-vulcanization is optimal for achieving the best thermal insulation performance. In addition, the reasonable thermal stability by 387.3 °C, flexibility as rubber, and the high E’ at 25 °C were observed in the PVNRL/SA_8-0_, supporting the ideal properties in thermal insulation and beneficial for further research in flexible thermal insulated thin-film applications, such as thin-film coating, and novel thermal insulated glove.

## 4. Materials and Methods

### 4.1. Materials

High-ammonia NRL was purchased with approximately 61.75% of total solid contents, and the vulcanizing agents, consisting of 60% sulfur dispersion, 50% zinc oxide (ZnO) dispersion, 50% zinc diethyldithiocarbamate (ZDEC) dispersion, and 50% LOWINOX™ CPL (CPL), were purchased from GSP Products Co., Ltd, Bangkok, Thailand. Potassium hydroxide (KOH) was procured from Suksapanpanit, Bangkok, Thailand. SA was received from RAGEL-P/REM TECH Co., Ltd, Deajon, Republic of Korea. The particle size, surface area, and bulk density were 10–200 nm, 300–350 m^2^ g^−1^, and 70–150 kg m^−3^, respectively. Ammonia buffer solution (pH 10) and polyethylene glycol sorbitan monolaurate (C_58_H_114_O_26_, Tween 20) were supplied by Sigma-Aldrich, MO, USA. Toluene (analytical grade) was bought from Merck, Darmstadt, Germany.

### 4.2. Pre-Vulcanization of NRL

The pre-vulcanization process began by stabilizing colloidal NRL with a KOH solution (10 wt%, 0.30 phr) to ensure uniform latex. The vulcanizing agents were added initially with sulfur dispersion (60 wt%, 0.50 phr), followed by stirring at 250 rpm for 5 min. Then, a ZnO suspension (50 wt% 0.50 phr), serving as an activator, was applied, and subsequently stirred at the same speed for 5 min. ZDEC (50 wt%, 0.75 phr) and CPL (50 wt%, 0.50 phr), acting as an accelerator and an antioxidant, respectively, were introduced, then stirred for 30 min at room temperature to obtain the compounded NRL. The stock PVNRL was prepared by maturing the obtained PVNRL for 0, 2, 4, 6, and 8 days at room temperature for the PVNRL/SA composites and the control sample preparation onward.

### 4.3. Preparation of SA Dispersion

The preparation of the SA dispersion was followed by previous work [32]. In brief, the premixed solution initially contained 100 g of deionized (DI) water and 0.40 g of Tween 20, serving as the medium and surfactant, respectively, and was stirred with a magnetic stirrer at 250 rpm for 15 min. The premixed medium was re-mixed with a 1500 W blender for 1 min, and then, 9.00 g of SA powder was added to the mixture and carefully blended. The cycle of blending steps lasted 1 min, followed by a 2 min pause, repeated a total of 5 times. The SA dispersion in an aqueous medium was adjusted to pH 7 with a 10 wt% KOH solution, and then the exact concentration of the SA dispersion was determined by total solid content investigation prior to composite preparation.

### 4.4. PVNRL/SA Composite Preparation

The PVNRL/SA composites were produced with various pre-vulcanization times from 0 to 8 days and SA contents of 20 phr. To prepare, PVNRL with different pre-vulcanization times, was initially mixed with 0.6 wt% ammonia buffer, then stirred at 250 rpm for 5 min. The obtained PVNRL was introduced to the SA dispersion during stirring. The mixture was continuously combined for 30 min, then left at room temperature for 1 h to eliminate the bubbles.

The PVNRL/SA slurries were cast onto a 0.5 mm thick glass plate and dried at room temperature (25 ± 3 °C) and 69 ± 4.5–humidity for 2 days, eventually obtaining the pre-vulcanized PVNRL/SA composites. The average thickness in 3 different positions was measured by a vernier caliper of 0.438 ± 0.055 mm. The dried composite films were stored in a refrigerator at 2 °C. The PVNRL films without SA were used as the control sample and prepared according to the mentioned process.

Additionally, to determine the effect of SA immersion on the PVNRL for confirming the mobility of NR chains through SA pores, the PVNRL/SA composites with different pre-vulcanization times (0, 4, 8 days) were stored in the refrigerator for 3 and 6 days before casting, and a similar drying process was then repeated at room temperature for 2 days.

### 4.5. Characterization and Testing

#### 4.5.1. Chemical Composition Analysis

The chemical composition of the PVNRL/SA composites and the control sample was studied by using Fourier transform infrared spectroscopy (FT-IR; PerkinElmer Scientific, Hopkinton, MA, USA). The spectra were collected between a wavenumber of 4000 and 400 cm^–1^, with the spectral resolution of 4 cm^–1^ and an accumulation time of 16 scans. The FT-IR intensity ratio for the C=C change was evaluated using three specimens per group. [35].

#### 4.5.2. Crosslink Density

PVNRL/SA composite and control samples, prepared by PVNRL with various pre-vulcanization times, were cut into rectangular specimens (0.05 g), precisely weighed (*W_u_*), and immersed in dried toluene (5 mL) within a glass bottle. These samples were maintained at 40 ± 3 °C for 48 h, with toluene replaced after 24 h to maintain equilibrium. Subsequently, the swollen samples were placed on filter paper to remove excess toluene from their surfaces. The samples were then weighed (*W_s_*) in a capped weighing bottle. The crosslink density (*ν*) for each composite and control sample was determined in triplicate, using the Flory–Rehner equation (Equation (1))

(1) $$\nu =\frac{-(\ln(1-V_{\;r}^{\;0})+V_{\;r}^{\;0}+\chi {V_{\;r}^{\;0}}^{2})}{2\rho_{r}\;V_{0}(V_{r}^{0^{1/3}}-\frac{V_{r}^{0}}{2})\;\;\;\;}$$

where $V_{\;r}^{\;0}$ represents the volume fraction of the vulcanized rubber, *χ* refers to the Huggins interaction constant, and *V*_0_ is the molar volume of the solvent used. The *V_r_*^0^ value was calculated using Equation (2).

(2) $$V_{\;r}^{\;0}={\left[\frac{\rho_{r}}{\rho_{s}}\;\text{×}\left(\frac{W_{s}-W_{u}}{W_{u}}\right)+1\right]}^{-1}$$

where *W_u_* and *W_s_* are the weights of the unswollen and swollen rubber samples at equilibrium, respectively. The *V*_0_ of toluene is 106.9 cm^3^ mol^−1^, and the *χ* constant for gum NR with toluene is 0.39. The densities of toluene (*ρ*_s_) and NR (*ρ*_r_) are 0.886 and 0.930 g cm^−3^, respectively [38]. Three parallel samples were tested for each group.

#### 4.5.3. Morphology

The morphologies of PVNRL, SA, and the PVNRL/SA composites were observed using a Field-emission scanning electron microscopy (FE-SEM; FEI, Quanta 250 FEG, Hillsboro, OR, USA). The micrograph was imaged with ×5K and ×100K magnification at an accelerating voltage of 10 kV, 9.7 mm of sample-lens distance, and the chamber pressure was maintained at 3 $\times 10^{-4}$–4 $\times 10^{-4}$ Pa. The PRVNRL/SA composites and PVNRL as control samples were fractured in liquid nitrogen, and then a thin layer of gold was coated for 3 min with 3 mA current using an ion sputter (JEC-1100E, JEOL Ltd., Tokyo, Japan) for all samples. To evaluate the elemental compositions, the PVNRL/SA composites were analyzed by energy dispersive X-ray spectroscopy (EDX; INCAx-act (Model 51-ADD0001), Oxford Instruments, Abingdon, UK). The pore area and diameter of the PVNRL/SA composite and the pristine SA were determined thanks to the ImageJ software, version 1.54 g. The investigated area in FE-SEM images was specified for 82.69 × 55.13 ${\mu m}^{2}\;$(×5K), and 4.12 × 2.75 ${\mu m}^{2}$ (×100K). The values obtained were averaged from two FE-SEM images for each sample [32].

#### 4.5.4. Thermal Conductivity

Thermal insulation performance of the PVNRL/SA composites and their control samples was indicated by thermal conductivity (k). All composite specimens were determined by utilizing a thermal conductivity analyser C-Therm TCi (C-Therm Technologies Ltd., Fredericton, NB, Canada). The operation of the instruments was carried out with the Modified Transient Plane Source technique. The heat generated by the applied electrical current raises the temperature at the interface between the sensor and the composite by approximately 2 °C, resulting in a voltage drop in the sensor element. The thermal conductivity was measured via the rate of the sensor’s compensated voltage with an accuracy of ±5% and a precision of ±1%. The thermal insulation of the composites is evident from the lower thermal conductivity value. Each sample was examined with three different runs at room temperature [20].

#### 4.5.5. Thermal Stability

Thermal stability of the PVNRL/SA composites was evaluated by a thermogravimetry analyzer (TGA; TG 209 F3 Tarsus, Netzsch, Selb, Germany). The TGA thermogram was recorded between 30 and 900 °C at 10 °C min^–1^ under a nitrogen atmosphere. In addition, the decomposition rate was analyzed simultaneously by differential thermal analysis (DTG) [19].

#### 4.5.6. Flexibility

Flexibility of the PVNRL/SA composites was studied using a dynamic mechanical analysis (DMA; DMA/SDTA1+, Mettler Toledo, Columbus, OH, USA). The rectangular specimens were prepared with dimensions of 8.5 mm in length, 4.5 mm in width, and 0.5 mm in thickness. The specimens were tested in tension mode with a scanning temperature range from −100 to 100 °C, at a heating rate of 2 °C/min, and a frequency of 1 Hz. The tanδ value resulting from E”/E’ was investigated for T_g_ indication. Each specimen was examined with two different runs [18,39].

## Figures and Tables

**Table 1:** Pore area and pore diameter of the PVNRL/SA composites with different pre-vulcanization and immersion times in 5K-FESEM images as the investigated area.

	Pore Area (%)	Pore Diameter (μm)
0-day PVNRL		
PVNRL/SA_0-0_	9.306 ± 3.551	5.703 ± 2.583
PVNRL/SA_0-3_	10.294 ± 3.434	5.068 ± 2.088
PVNRL/SA_0-6_	10.295 ± 0.108	5.189 ± 2.841
4-day PVNRL		
PVNRL/SA_4-0_	20.873 ± 0.463	6.321 ± 3.583
PVNRL/SA_4-3_	17.606 ± 0.751	5.561 ± 2.855
PVNRL/SA_4-6_	16.488 ± 1.193	4.875 ± 3.064
8-day PVNRL		
PVNRL/SA_8-0_	23.391 ± 2.001	6.175 ± 3.832
PVNRL/SA_8-3_	21.513 ± 0.877	7.108 ± 6.792
PVNRL/SA_8-6_	16.771 ± 0.229	6.483 ± 3.692

**Table 2:** Pore area and pore diameter of the PVNRL/SA composites with different pre-vulcanization and immersion times in 100K-FESEM images as the investigation area.

	Pore Area (%)	Pore Diameter (μm)
0-day PVNRL		
PVNRL/SA_0-0_	0.270 ± 0.047	0.062 ± 0.004
PVNRL/SA_0-3_	0.581 ± 0.068	0.089 ± 0.028
PVNRL/SA_0-6_	1.068 ± 0.108	0.106 ± 0.004
4-day PVNRL		
PVNRL/SA_4-0_	19.198 ± 1.999	0.120 ± 0.034
PVNRL/SA_4-3_	16.877 ± 3.511	0.157 ± 0.015
PVNRL/SA_4-6_	18.046 ± 2.259	0.168 ± 0.023
8-day PVNRL		
PVNRL/SA_8-0_	29.480 ± 7.623	0.090 ± 0.034
PVNRL/SA_8-3_	14.853 ± 1.006	0.135 ± 0.007
PVNRL/SA_8-6_	18.407 ± 1.374	0.121 ± 0.042
SA	32.113 ± 7.484	0.091 ± 3.692

## Data Availability

The original contributions presented in this study are included in the article. Further inquiries can be directed to the corresponding author.

## Abbreviations

The following abbreviations are used in this manuscript:

PVNRL	Pre-vulcanized natural rubber latex
SA	Silica aerogel
NRL	Natural rubber latex
RPs	Rubber particles
FT-IR	Fourier transform infrared spectroscopy
FE-SEM	Field-emission scanning electron microscopy
EDX	Energy dispersive X-ray spectroscopy
TGA	Thermogravimetry analysis
DTG	Differential thermal analysis
DMA	Dynamic mechanical analysis
DI	Deionized
T_d_	Decomposition temperature
Tan δ	Loss tangent
T_g_	Glass transition temperature
